# Treating hot flushes with a neurokinin 3 receptor antagonist

**DOI:** 10.18632/oncotarget.22383

**Published:** 2017-11-11

**Authors:** Julia K. Prague, Waljit S. Dhillo

**Affiliations:** Waljit S. Dhillo: Department of Investigative Medicine, Imperial College London, London, UK

**Keywords:** neurokinin 3 receptor, hot flushes, RCT

Seventy percent of women are affected by hot flushes. The majority describe them as the most bothersome symptom that they experience during the menopause [1] as they are typically long-lasting (median 7.4 years) [2] and impact on many aspects of their daily life. Such symptoms are a consequence of falling oestrogen levels as the remaining number of ovarian follicles decline. As such an effective treatment is Hormone Replacement Therapy which restores circulating oestrogen levels. However HRT, and in particular combined preparations with oestrogen and progesterone, are not without risk, and are contraindicated in many women; for example those with a prior history of breast cancer. Other alternative therapies such as some anti-depressants, gabapentin, herbal remedies, and cognitive behavioural therapy have been shown to have some efficacy over placebo but are also not without side-effects and/or may not be widely available. A novel therapeutic that was effective and safe could therefore benefit a huge number of women, with an estimation of 10 million women in the UK alone [3]. However in order to achieve this the aetiology of hot flushes needed to be better understood.

Over the last 20 years, Naomi Rance and colleagues have progressed the scientific understanding of the aetiology of menopausal hot flushes using human brain tissue obtained from post-mortems and animal models (monkey and rat). They have shown that menopausal hot flushes occur due to upregulation in the signalling of neurokinin B (NKB), a hypothalamic neuropeptide, in association with its receptor (neurokinin 3 receptor; NK3R) in response to oestrogen deficiency via the median pre-optic nucleus, which receives input from, and projects to, the autonomic thermoregulatory pathway [4, 5]. In keeping with this, an early randomised, placebo-controlled, clinical trial by our group demonstrated that peripheral intravenous infusion of NKB induced hot flushes in pre-menopausal women that were very similar to those experienced by menopausal women [6].

Based on these data we hypothesised that an oral, NK3R antagonist would attenuate menopausal hot flushes. We designed a randomised, double-blind, placebo-controlled, crossover trial to test this in a proof-of-concept study, which published earlier this year [7]. In our investigator initiated and MRC/NIHR funded study, we recruited women aged between 40 and 62 years who had at least seven hot flushes/24 hour period, some of which had to be severe or bothersome, and who had not menstruated for at least 12 months. Participants were randomised to receive either four weeks of treatment with an oral NK3R antagonist twice daily (MLE4901, Millendo Therapeutics, Inc., Ann Arbor, US) or exact-match placebo twice daily. Following this all participants then entered a two week washout phase, and then switched to have the second intervention that they had not yet received previously. Participants recorded their symptoms in real-time, completed daily questionnaires, and for the first 48 hours of each week wore a skin conductance monitor on their sternum to objectively measure hot flushes. They also attended for a weekly review where blood samples were taken to measure hormone concentrations and renal/liver function for safety monitoring.

MLE4901 significantly reduced the total weekly number of hot flushes by 45 percentage points compared to placebo. Furthermore, compared to baseline, hot flush frequency reduced by 73%, severity by 45%, bother by 51%, and interference by 72% after four weeks of treatment with the oral NK3R antagonist. Good concordance was shown between subjective reporting and objective measurement of hot flushes confirming accuracy of our results. This is the first report in humans that an oral NK3R antagonist can effectively attenuate menopausal hot flushes without the need for oestrogen exposure or an increase in circulating oestradiol concentrations by preventing propagation of the NKB signal.

MLE4901 was also well tolerated, and no serious adverse events occurred. Three participants did develop a transient and asymptomatic rise in liver transaminases with a normal bilirubin following 28 days treatment with MLE4901, and this will need to be investigated further in larger subsequent trials.

Our findings fit entirely with the pre-existing data and suggest great promise for this therapeutic class in the future treatment of menopausal hot flushes [8]. Furthermore, as we believe the mechanism for alleviating hot flushes to be the same in the menopause as for women taking oestrogen deprivation therapy for breast cancer treatment, and for men taking androgen deprivation therapy for prostate cancer treatment this could therefore be a novel agent that could bring hope to many cancer sufferers as it does not require sex steroid exposure. Further larger and longer studies in menopausal women and cancer patients are required to ensure that efficacy and safety is confirmed in all potential therapeutic groups but if this is shown to be the case then NK3R antagonists could be truly practice-changing.
